# Development of a Desmocollin-3 Active Mouse Model Recapitulating Human Atypical Pemphigus

**DOI:** 10.3389/fimmu.2019.01387

**Published:** 2019-06-19

**Authors:** Roberta Lotti, Claudio Giacinto Atene, Alessandra Marconi, Giulia Di Rocco, L. Reggiani Bonetti, Tommaso Zanocco Marani, Carlo Pincelli

**Affiliations:** ^1^Laboratory of Cutaneous Biology, Department of Surgical, Medical, Dental and Morphological Sciences, University of Modena and Reggio Emilia, Modena, Italy; ^2^Department of Life Sciences, University of Modena and Reggio Emilia, Modena, Italy; ^3^Department of Medical and Surgical Sciences of Children & Adults, University of Modena and Reggio Emilia, Modena, Italy

**Keywords:** pemphigus, mouse model, desmocollin-3, desmoglein-3, autoimmunity

## Abstract

Pemphigus vulgaris (PV) is a life-threatening mucocutaneous autoimmune blistering disease. It is often associated with autoantibodies to the desmosomal adhesion proteins Desmoglein 3 (DSG3) and Desmoglein 1 (DSG1). Recently, auto-antigens, such as desmocollins and others have been described in PV and in atypical pemphigus forms such as Pemphigus Herpetiformis (PH), Pemphigus Vegetans (PVeg), and Paraneoplastic Pemphigus (PP). Desmocollins belong to a cadherin subfamily that provides structure to the desmosomes and play an important role in cell-to-cell adhesion. In order to verify the pathogenic activity of anti-Desmocollin 3 (DSC3) antibodies, we developed an active disease model of pemphigus expressing anti-DSC3 autoantibodies or anti-DSC3 and anti-DSG3 antibodies. This approach included the adoptive transfer of DSC3 and/or DSG3 lymphocytes to Rag2^−/−^ immunodeficient mice that express DSC3 and DSG3. Our results show that the presence of anti-DSC3 auto-antibodies is sufficient to determine the appearance of a pathological phenotype relatable to pemphigus, but with features not completely super-imposable to those observed in the DSG3 active model, suggesting that the DSC3 active model might mimic the atypical pemphigus. Moreover, the presence of both anti-DSC3 and anti-DSG3 antibodies determines a more severe phenotype and a slower response to prednisolone. In conclusion, we have developed an adult DSC3 pemphigus mouse model that differs from the DSG3 model and supports the concept that antigens other than desmogleins may be responsible for different phenotypes in human pemphigus.

## Introduction

The term pemphigus gathers together a group of chronic life-threatening autoimmune disorders characterized by blistering of the skin and of mucous membranes. Blistering is caused by loss of cell-to-cell adhesion (acantholysis) determined by the presence of autoantibodies targeting the surface of keratinocytes. Traditionally, pemphigus is classified in two main forms: pemphigus vulgaris (PV) and pemphigus foliaceus (PF), depending on the epidermal layer where blistering occurs. Seminal studies identified desmosomal proteins Desmoglein3 (DSG3) and Desmoglein1 (DSG1) as the primary targets of autoantibodies in PV (1–3). However, recent studies suggest that the presence of autoantibodies against DSG1 and DSG3 alone is not sufficient to fully explain the loss of cell-to-cell adhesion observed in pemphigus (4). Moreover, non-Desmoglein antigens and autoantibodies have been detected in PV and other forms of pemphigus, such as atypical pemphigus (4, 5). These forms of pemphigus are characterized by the absence of Desmoglein autoantibodies and by the presence of different non-DSG autoantibodies that might act in synergy to develop the pathological phenotype of atypical pemphigus (6). Non-Desmoglein antigens observed in PV and in atypical pemphigus (5), include cadherins, cholinergic receptors, mitochondrial proteins, and members of the armadillo family of proteins. In particular, Desmocollins that are responsible for the structure of the desmosome (7) and play a fundamental role in cell-to cell adhesion (8, 9) seem to be particularly relevant. Indeed, DSC1 and DSC3 auto-antibodies have been found in several forms of atypical pemphigus, in the absence or- less frequently- in the presence of anti-DSG autoantibodies (10, 11).

Several animal models have been made available for PV. The first passive animal model (12) was obtained by inoculating neonatal mice with IgG fractions obtained by patients' sera. A modified version of this passive transfer model was obtained by inoculating adult mice with hybridoma cells producing monoclonal anti-DSG3 antibodies (13). Active disease models have been developed for PV and have shown to be useful tools to evaluate therapeutic strategies that target Desmoglein 3-reactive T cells and B cells (13, 14). By this approach, DSG3 knockout mice were used to generate an immune response against DSG3 and produce anti-DSG3 IgG antibodies. Adoptive transfer of peripheral lymphocytes from Dsg3^−/−^ mice to immune-deficient but Desmoglein 3-expressing recipient mice generated an artificial autoimmune state in the recipient mice (14). This active disease model of pemphigus vulgaris has proved to be useful to evaluate the efficacy of pharmacological reagents as well as cell therapies to block antibody production (15). In general, active models seem to be more representative of pemphigus than passive models, since they allow to observe the pathological phenotype through the entire lifespan of the animal.

To better understand the pathogenic role of non-DSG autoantibodies, here we report the first DSC3 active pemphigus mouse model. In addition, we present a new active model expressing both anti-DSC3 and anti-DSG3 antibodies. We show that DSC3 mouse recapitulates aspects of the human atypical pemphigus, and that DSC3/DSG3 mice express a more severe phenotype that fail to respond to systemic steroids.

## Methods

### Cell Cultures

Sf9 insect cells (Gibco, Rockville, MD, USA) were cultured in Insect-XPRESS Protein-free Insect Cell Medium with L-Glu (Lonza, Walkersville, MD), containing streptomycin (100 μg/mL) and penicillin (100 U/mL) (Gibco, Rockville, MD, USA), in serum free conditions (FBS, Lonza, Walkersville, MD). Adherent cultures were maintained at 27°C without the need of CO_2_-humidified atmosphere. Sf9 cells adapted to suspension culture were maintained on a shaker apparatus between 125 and 150 rpm in a non-humidified incubator at 27°C.

### Recombinant Protein Expression

**Figure 1:**
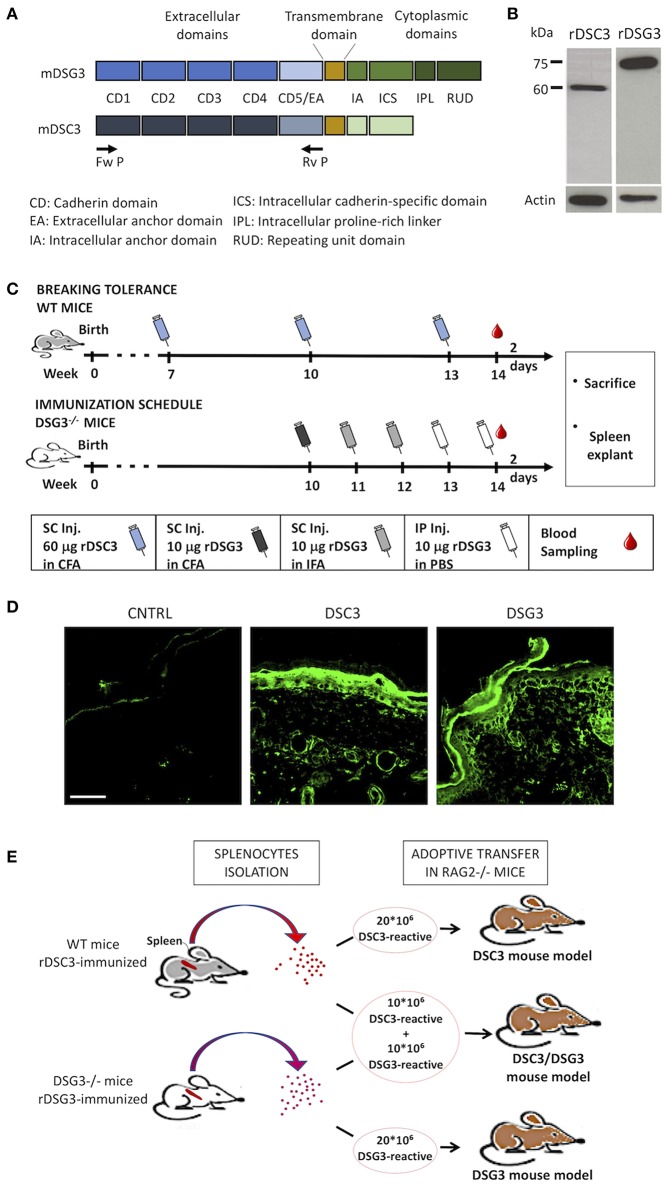
Production of mouse recombinant DSG3 and DSC3 and immunization scheme of mice. **(A)** Scheme of the recombinant proteins used in this study, i.e., the entire extracellular domains of murine DSG3 (mDSG3, 12) and murine DSC3 (mDSC3) were cloned and linked to 6xHis-Tag. **(B)** Detection of the recombinant proteins in Sf9 cell lysates by immunoblot analysis using an anti-His-tag monoclonal antibody. Actin was used as loading control. **(C)** Immunization schemes used for DSC3 breaking tolerance protocol in WT mice and for DSG3 in Dsg3^−/−^ mice. CFA, Complete Freund's Adjuvant; IFA, Incomplete Freund's Adjuvant. **(D)** Indirect Immunofluorescence of immunized animal sera on WT neonatal mouse skin sections. CNTRL: Serum from animals immunized with non-infected Sf9 cells proteins emulsified in FCA. Scale bar: 50 μm. **(E)** Schematic representation of the adoptive transfer protocol in mice.

Mouse Desmocollin 3 was expressed as 6xHis-tag fusion protein in Sf9 insect cells as follows. Total cDNA was generated by RT-PCR performed on total RNA extracted from mouse skin biopsy. The extracellular portion of mDSC3 was generated by PCR using the following primers: FW 5′-ATGGAGCACAAGAAGAAGGTACTGA-3′ and RV 5′-AAGGATGGCCCACTTCCCAG-3′ (Figure 1A). The PCR product was subcloned in the pFastBac/C-His TOPO vector (Bac-to-Bac C-His TOPO® cloning and expression Kit, Invitrogen, Carlsbad, CA) with a C-terminal His-tag. The recombinant clones were analyzed by restriction enzyme digestion and sequencing of the DSC3 insertion. DSC3 clones were then transformed into a competent DH10Bac *E. coli* strain. Colonies that contain recombinant bacmid were verified by PCR analysis with the pUC/M13 forward and reverse primers. The recombinant bacmid DNA was transfected in Sf9 cells using Cellfectine II reagent (Invitrogen, Carlsbad, CA) following manufacturer's instructions. Cells were incubated at 27°C from 4 to 6 days. When cells showed signs of viral infection the medium was collected and centrifuged, and the supernatant was stored at 4°C as the P0 stock. After several round of infections, we collected the P3 stock.

On the other hand, the P3 baculoviral stock for rDSG3 production was a kind gift of Dr. Amagai. This vector allows active secretion of recombinant 6xHis-tagged protein in culture medium and was constructed as described in Amagai et al. (14).

Protein expression was carried on in suspension condition, in shaking flask incubated at 27°C with shaking at 135 rpm. 2–4^*^10^6^/mL Sf9 cells were infected with concentrated P3 recombinant baculoviral stock at MOI 5.

### Recombinant Protein Purification

For the rDSC3 production, the insect cells were collected by centrifugation 120 h post-infection and resuspended in ice-cold lysis buffer (50 mM NaH_2_PO_4_, 250 mM NaCl, 1 mM CaCl_2_ with 0,1 mM PMSF, 0.1% Triton-X100, 5 U/mL Benzonase, pH 7.8). The sample was centrifuged at 40,000x g for 30' and the supernatant was loaded on the 5 mL HisTrap FF crude column (GE Healthcare, Little Chalfont, UK) after addition of 20 mM of imidazole. By Akta Prime chromatographic system (GE Healthcare, Little Chalfont, UK), rDSC3 was purified using 10 column volume of wash buffer (containing 10 mM of additional imidazole) and a linear gradient of 16 column volume of elution buffer (containing a total concentration of 300 mM of imidazole). In each step, 8 mL fractions were collected for SDS-PAGE and western blot analysis (Supplementary Figure 1A). The purified protein was dialyzed with cellulose membranes (cut-off 10 kDa) (Sigma Aldrich, Saint Louis, MO) in 50 mM NaH_2_PO_4_, 250 mM NaCl, 1 mM CaCl_2_, pH 7.8 to eliminate imidazole. Fractions with rDSC3 were pooled, quantified and lyophilized.

The rDSG3 was purified from cell culture medium. 1 L of medium was concentrated with ultra-filtration discs (cut-off 10 kDa) on amicon stirred cell (Millipore, Burlington, MA). The concentrate medium was dialyzed as described before. After two runs of dialysis, 10 mM of imidazole was added to the sample that was subsequently loaded in 5 mL HisTrap FF crude column. Purification and collection step were the same performed for rDSC3.

### SDS-Page and Western Blot

To verify the protein production in test expression experiment, infected cells were harvested, washed with PBS and lysed on ice in RIPA buffer containing protease inhibitors. 30 μg of total protein were analyzed on polyacrylamide gels and blotted onto nitrocellulose membranes. Blots were blocked for 1 h in blocking buffer (5% non-fat milk in PBS/0.05% Tween20) and incubated minimum 4 h or overnight at 4°C with the mouse anti-6x-HisTag monoclonal antibody (3D5; Invitrogen, Carlsbad, CA) and anti-actin monoclonal antibody (C2; Santa Cruz Biotechnology, Dallas, TX). Then membranes were washed in PBS/0.05% Tween20, incubated with HRP-conjugated goat anti-mouse antibody (B2213; Santa Cruz Biotechnology, Dallas, TX) for 1 h at room temperature. After washing steps, membranes were developed using the ECL chemiluminescent detection system (Amersham Biosciences, Little Chalfont, UK).

Western blot analysis of fractions collected during rDSC3 and rDSG3 purification was performed by loading 40 μl of samples per each fraction (Supplementary Figures 1A,B).

### Mice Immunization

B6;129X1-Dsg3tm1Stan/J and B6(Cg)-Rag2tm1.1Cgn/J adult mice were obtained from Charles River Italia (Calco, Italy) and maintained at the Laboratory of Animal Facility, University of Modena and Reggio Emilia. Dsg3^−/−^ offspring were obtained by mating male and female Dsg3+/– mice. For induction of an autoimmune response to murine rDSG3, mice were immunized as illustrated in Figure 1C. 10 weeks-old Dsg3^−/−^ mice were primed with subcutaneous injection of 10 μg rDSG3 emulsified in Imject Complete Freund's Adjuvant (CFA) (Thermo Scientific, Waltham, MA). After 1 and 2 weeks, mice were boosted with subcutaneous injection of 10 μg rDSG3 emulsified with Imject Incomplete Freund's Adjuvant (FIA) (Thermo Scientific, Waltham, MA). Then, other two injections of 10–20 μg rDSG3 were performed weekly intraperitoneally without adjuvant. During last immunization, a blood sample was collected to verify the presence of circulating anti-DSG3 IgG. To obtain the reactive splenocytes, mice were sacrificed 30 days after first immunization.

Immunization protocol leading to the loss of tolerance to DSC3 was performed in WT mice (Figure 1C) slightly modifying the protocol described by Hirose et al. (16). 7-weeks-old WT mice were immunized at the hind footpad with 60 μg rDSC3 emulsified in Imject Complete Freund's Adjuvant (CFA). Mice were further immunized 3 and 6 weeks after initial immunization in the same manner. During last immunization, a blood sample was collected to verify the presence of circulating anti-DSC3 IgG. To obtain the reactive splenocytes, mice were sacrificed 51 days after first immunization.

Negative controls (CNTRL animals) were generated by immunization with the sample resulting from non-infected Sf9 cells proteins emulsified in complete Freund's adjuvant, following the breaking tolerance protocol.

### Adoptive Transfer of Splenocytes

Splenocytes were isolated from immunized Dsg3^−/−^ or WT mice by disrupting their spleen and lysing the erythrocytes. Usually, the splenocytes were pooled from two or more immunized Dsg3^−/−^ or WT mice. 20^*^10^6^ DSC3 or DSG3-reactive splenocytes were transferred via tail vein injection in 10–11-weeks-old Rag2^−/−^ mice. To develop the DSC3/DSG3 mouse model, 10^*^10^6^ DSC3-reactive splenocytes and 10^*^10^6^ DSG3-reactive splenocytes were injected per mouse (Figure 1E).

### Administration of Methyl-Prednisolone to Pemphigus Model Mice

Methyl-prednisolone (m-PSL) (Solu-Medrol® Pfizer, Tokyo, Japan) was given intraperitoneally at a dose of 100 mg/kg daily (15), starting from day 7 after adoptive transfer and continued for 4 weeks, till day 35. The mice were evaluated for 9 weeks.

### Indirect Immunofluorescence

Sera of immunized mice were analyzed by indirect immunofluorescence microscopy on cryosections of WT murine skin. Sections were blocked with 1% bovine serum albumin and 1% goat normal serum for 20', and then incubated with sera (1:50) for 1 h, and finally for 45' with Alexa Fluor 488 anti-mouse antibody (Invitrogen Corporation, Carlsbad, CA, USA). Fluorescent specimens were analyzed by a Confocal Scanning Laser Microscopy (Leica TCS SP2).

### Pemphigus Phenotype Scoring

To evaluate the level of disease in the pemphigus mouse models, PV score was estimated weekly by counting the number of affected sites (Table 1). Briefly, we slightly modified the Aoki Ota evaluation table (17), counting a score of 1 for erosion, 0.5–1 for hair loss, and 0.5–1 for erythema. The maximum total scores for erosive lesions, hair loss, and erythema was 16. When mice died, the PV scores at the time of death were used as virtual scores thereafter. In addition to the PV score, each week the weight of the animals was recorded in order to report the weight loss from baseline (*t* = 0, corresponding to the moment of splenocytes transfer).

**Table 1:** Summary of the frequency of the phenotypic aspects presented by the three pemphigus mouse models.

**Type of lesion**	**Sites**	**DSC3 (%)**	**DSC3/DSG3 (%)**	**DSG3 (%)**
Erosion (1)	Muzzle	28	83	78
	Periocular region	–	50	33
	Periauricular region	–	25	22
	Back	–	8	11
	Chest	9	–	22
	Abdomen	–	–	–
	Right foreleg	9	33	22
	Left foreleg	18	25	11
	Right hind leg	–	–	11
	Left hind leg	–	8	11
	Tail	9	58	33
Alopecia	Face (1)	91	100	100
	Neck (0.5)	91	100	100
	Back (1)	73	100	56
	Abdomen (0.5)	100	83	100
Erythema (1)	Footpad	100	100	56
	Abdomen	91	83	22

*In brackets the score for each symptom*.

### Histology Analysis

For each sample a representative portion containing both normal and lesional skin was fixed in 4% neutral buffered formalin and paraffin embedded. Skin sections were analyzed by hematoxylin and eosin (H&E) staining. For evaluation of eosinophils, we performed Pagoda Red (Dylon International LTD, England) staining (18). Samples were analyzed using a conventional optical microscope (Zeiss Axioskope 40). Five random microscopic fields per sample were captured at 200X magnification. AxioVision AC imaging software was used to acquire sample images.

### Mouse DSC3 and DSG3 ELISA Assay

Not coated microtiter 96-well plates were coated with 50 μL of 4 μg/mL purified murine rDSC3 or rDSG3 at 4°C overnight. The plates were washed with washing buffer (PBS with 0.1% Tween20, pH 7.3) and then incubated in blocking buffer (PBS with 0.1% Tween20 and 5% BSA) at 4°C overnight. Sample sera were diluted 200-fold and incubated for 1 h at room temperature on coated plates. After washing three times with washing buffer, plates were then incubated with HRP-conjugated goat polyclonal secondary anti-mouse IgG antibody (1:3,000; AbCam, Cambridge, UK) for 1 h at room temperature. Three additional wash were performed. Then the TMB substrate solution was added and incubated for 30' at room temperature. The reaction was stopped by adding stop solution, containing1 mol/l H_2_SO_4_. The absorbance was measured at 450 nm, with reference at 620 nm, by iMark™ Microplate Absorbance Reader (BioRad, Hercules, CA, USA). DSC3- or DSG3-immunized mouse sera were used as positive controls, while serum from WT (not immunized) mice was used as negative control. The index value was defined by the following formula: index = (optical density [OD] of tested serum – OD of negative control)/(OD of positive control – OD of negative control) × 100. When the ELISA index values were >200, the sera were further diluted, and the final index values were obtained by multiplying the index values by the dilution factor.

### Ethics Statement

Animal studies and animal procedures were approved by the Animal Welfare Committee of the University of Modena and Reggio Emilia and carried out in accordance with the Italian Institute of Health guidelines. The protocol was approved by the Italian Ministry of Health.

### Statistical Analysis

Data are presented as mean ± SEM, obtained from three to five different experiments. Prism Software (Graph Pad Software V8.0) was used to perform statistical analysis. A two-tailed unpaired Student's *t*-test was used for statistical comparisons between two groups, while one-way or two-way ANOVA was used for multiple comparisons. Usually ANOVA was associated to Multiple *t*-test analysis (as indicated in figure legends). A value of *P* < 0.05 or less was assumed to indicate a statistically significant difference in the compared parameters.

## Results

### Development of Active DSC3 and DSG3/DSC3 Pemphigus Mouse Models

Extracellular domains of the murine DSG3 and DSC3 were cloned (Figure 1A) and both recombinant DSG3 (rDSG3) and DSC3 (rDSC3) were produced by insect cells infected by baculovirus as described in the methods section. In particular, both rDSG3 and rDSC3 correspond to the entire NH_2_-terminal portion of the murine protein, encompassing all Cadherin Domains (CD1-CD2-CD3-CD4-CD5) and the Extracellular Anchor domain, against which auto-Abs are generated. Proteins were purified by HisTrap FF column with a gradient from 20 to 3,000 mM imidazole. For both rDSG3 and rDSC3 no contaminating proteins were found (Supplementary Figures 1A,B). The presence of rDSG3 and rDSC3 in the eluate was confirmed by western blot (Figure 1B).

The protocols for immunizing Dsg3^−/−^ mice with rDSG3 (12) and to break immunological tolerance to DSC3 in wild type (WT) mice (modified from 16) are schematically presented (Figure 1C). In particular, WT mice were immunized with 60 μg rDSC3 emulsified in complete Freund's adjuvant 7 weeks following birth. They were then boosted at weeks 10 and 13. At week 14 mice were sacrificed, their spleen was explanted and the presence of antibodies in their serum was evaluated by indirect immunofluorescence using mouse WT skin as a substrate (Figure 1D). Anti-DSG3 is present mainly in the basal and immediately suprabasal layers of the epidermis. Anti-DSC3 display the same kind of expression, as described in literature (19), and consistent with the pattern observed in human skin (20). After confirming the presence of antibodies, splenocytes were isolated from rDSG3 immunized DSG3^−/−^ mice and from rDSC3 immunized WT mice. To develop the DSG3 and the DSC3 single antigen models, 20 × 10^6^ splenocytes were injected in Rag2^−/−^ mice, while, to develop the mixed antigen model (DSC3/DSG3), 10 × 10^6^ for each type of reactive splenocytes were injected in Rag2^−/−^ mice (Figure 1E).

### Phenotypic, Histopathological, and Immunological Evaluation of Pemphigus Models

**Figure 2:**
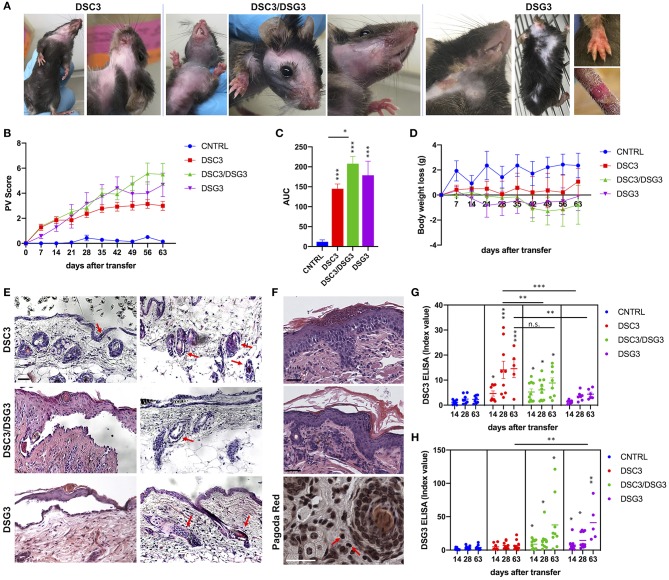
Phenotypic, histopathological, and immunological evaluation of the three pemphigus mouse models. **(A)** Phenotype of active pemphigus mouse models. Mice injected with DSC3 autoreactive splenocytes developed crusted erosions around the snout and cheeks. Intense erythema and patchy hair loss are the main phenotypic aspects. Animals receiving DSC3/DSG3 autoreactive splenocytes exhibit a more severe phenotype, with crusted erosions also on paws, large areas of alopecia and exacerbated erythema. DSG3 animals perfectly reproduce the phenotype formerly published (12). **(B)** Phenotypic aspects were translated into PV score, weekly evaluation, with an observational period of 63 days after splenocytes transfer into Rag2^−/−^ mice. (*n* = 7–9 animals). Two-way ANOVA test between treatments is highly significant, *P* < 0.0001. DSG3/DSG3 vs. CNTRL *P* = 0.0137, DSG3 vs. CNTRL *P* = 0.0293, from day 21. DSC3 vs. CNTRL *P* = 0.0104, from day 35. **(C)** PV score overtime translated in Area Under the Curve (AUC). One-way ANOVA between treatments, *P* < 0.0001. All treated animals are significantly different from CNTRL, as shown. Moreover, Unpaired *t*-test between DSC3 vs. DSC3/DSG3, *P* = 0.0107. **(D)** Body weight loss from the baseline (*t* = 0, splenocytes infusion time) overtime (weekly evaluation, 63 days after splenocytes transfer). DSC3/DSG3 vs. CNTRL *P* = 0.0479, from day 49. **(E)** Histologic examination of active mouse models by H&E. Scale bar: 50 μm. **(F)** Higher histologic magnification of DSC3/DSG3 mouse skin with spongiosis (upper panel), dermal infiltrate (middle panel) and eosinophils (Pagoda Red, lower panel). Scale bar: 50 μm. **(G)** DSC3 ELISA. Two-way ANOVA between treatments. Only DSC3 and DSC3/DSG3 titers are significantly modulated against CNTRL at each time-point. Moreover, DSC3 is statistically different also vs. DSG3 at each timepoint (*P* = 0.02). **(H)** DSG3 ELISA. Two-way ANOVA between treatments. DSC3/DSG3 and DSG3 titers significantly modulated against CNTRL at each timepoint. Moreover, DSC3/DSG3 and DSG3 are significantly different also vs. DSC3 at each timepoint (i.e., *P* = 0.0004, day 63). ^*^0.05 < *p* < 0.01; ^**^p < 0.01; ^***^p < 0.0001.

Rag2^−/−^ mice that received DSG3^−/−^ splenocytes spontaneously developed a pemphigus vulgaris phenotype, with erosions and alopecia, as previously described (14). Rag2^−/−^ mice that received splenocytes deriving from WT mice immunized with rDSC3 in association with splenocytes from DSG3^−/−^ mice developed a more severe phenotype, while Rag2^−/−^ mice receiving only splenocytes obtained from WT animals immunized with rDSC3 spontaneously developed features suggestive of the human form of “atypical pemphigus.” The phenotypic aspects expressed by the three different mouse models are summarized in Table 1. As expected, DSG3 animals are more prone to develop erosions and blisters in different body areas. Interestingly all DSC3 transfused Rag2^−/−^ mice develop pemphigus mouse features, with erythema and alopecia. DSC3/DSG3 model presents characteristics of both DSG3 and DSC3 driven phenotypes. In particular, DSG3 animals exhibit erosions, blisters and patchy hair loss on the snout, face, chest, legs and tail. DSC3/DSG3 mice show more lesions and alopecia on the face and in the periocular area, erosions on the forelegs, with exacerbated erythema, while in DSC3 mice intense erythema and patchy hair loss are the main phenotypic aspects. These mice also display crusted erosions mainly localized around the snout and cheeks, where they normally scratch (Figure 2A). Altogether, the two pemphigus models reacting against DSG3 have a higher PV score, as compared to the DSC3 mice (Figure 2B). By the analysis of the Area Under the Curve (AUC), all treated animals have a significantly higher PV score than animals receiving CNTRL splenocytes. Moreover, score in DSC3 mice is statistically lower than in DSC3/DSG3 mice, *P* = 0.0107 (Figure 2C). To further characterize pemphigus mice, we evaluated the body weight over time. DSC3/DSG3 and DSG3 mice undergo a more pronounced weight loss, as compared to the DSC3 animals, confirming a more severe phenotype. In particular, DSG3/DSC3 mice experience the most relevant weight loss, reaching a plateau at 49 days after splenocyte transfer (Figure 2D). The histologic examination revealed an extensive intraepithelial loss of cell-cell adhesion just above the basal layers (suprabasal acantholysis) in the epidermis and hair follicles of the two mouse models reacting against DSG3. On the other hand, in DSC3 models, acantholysis is visible at a lower extent (see arrows, Figure 2E). Cleft formation between the cells surrounding the telogen club and the basal layer of the outer root sheath epithelium (arrows) and empty, dilated telogen hair follicles (arrows) account for the massive alopecia observed in the three models (Figure 2E). Only in the DSC3/DSG3 mouse model, high magnification histological analysis revealed epidermal focal spongiosis and some inflammatory cells infiltrating the dermis, in presence or absence of crusted lesions (Figure 2F). Scattered neutrophils were found in crusted lesions, but it is to be determined if they are secondary to scratching. Finally, some occasional eosinophils were detected by Pagoda Red staining (see arrows, Figure 2F). The production of autoreactive IgG was analyzed 14, 28, and 63 days after splenocyte transfer (Figures 2G,H). Using DSC3 ELISA assay, titers of DSC3 and DSC3/DSG3 antibodies are significantly increased against CNTRL at each timepoint. Moreover, antibody titers in DSC3 model is statistically higher than in DSG3 mice, at each timepoint (*P* = 0.02) (Figure 2G). On the other hand, by DSG3 ELISA, titers in DSC3/DSG3 and DSG3 mice are statistically increased against CNTRL at each timepoint. Moreover, DSC3/DSG3 and DSG3 titers are statistically different also from DSC3 at each timepoint (i.e., *P* = 0.0004, day 63) (Figure 2H).

### Effect of Methyl-Prednisolone (m-PSL) in the Three Pemphigus Mouse Models

**Figure 3:**
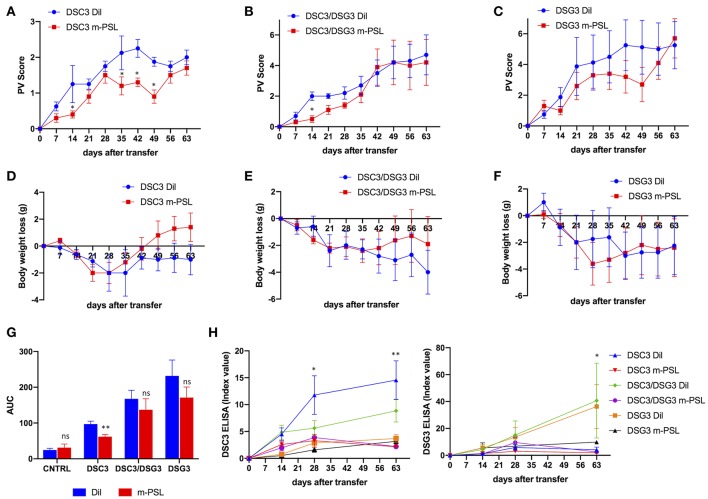
Effect of methyl-prednisolone (m-PSL) treatment in the three pemphigus mouse models. m-PSL was administered i.p. daily from day 7 after the adoptive transfer to day 35. Animals were randomly assigned to the m-PSL or PBS (Dil) treatment group (*n* = 5–7 animals per group). PV score **(A–C)** and body weight variations **(D–F)** were reported weekly, till day 63. For DSC3 mouse model: **(A)** PV score: by multiple *t*-test at each timepoint, m-PSL is statistically different from Dil at day 14 (*P* = 0.048), day 35 (*P* = 0.028), day 42 (*P* = 0.024) and day 49 (*P* = 0.021); **(D)** Body weight loss: no statistically significant differences among m-PSL and Dil group. For DSC3/DSG3 mouse model: **(B)** PV score: by multiple *t*-test at each timepoint, m-PSL is statistically different from Dil only at day 14 (*P* = 0.025); **(E)** Body weight loss: no statistically significant differences among m-PSL and Dil group. For DSG3 mouse model: **(C)** PV score and **(F)** Body weight loss no statistically significant differences among m-PSL and Dil group. **(G)** PV score overtime translated in Area Under the Curve (AUC). Two-way ANOVA between conditions, *P* < 0.0001. By Multiple *t*-test analysis, m-PSL group is statistically different from Dil group only in DSC3 model (*P* = 0.01). **(H)** DSC3 and DSG3 ELISA assay. Two-way ANOVA between conditions, *P* < 0.0001 for DSC3 analysis and *P* = 0.0076 for DSG3 analysis. In DSC3 ELISA, m-PSL significantly suppressed the production of anti-DSC3 IgG starting from day 28 in DSC3 model, and at day 63 in DSC3/DSG3 mouse model (detailed in Supplementary Figure 3). In DSG3 ELISA, m-PSL significantly suppressed the production of anti-DSG3 IgG at day 63 in DSC3/DSG3 and DSG3 mouse models (detailed in Supplementary Figure 3). ^*^0.05 < *p* < 0.01; ^**^p < 0.01.

In order to further characterize and compare the three pemphigus mouse models, all animals underwent treatment with m-PSL, a well-known glucocorticoid used in the therapy of pemphigus. 100 mg/Kg m-PSL or PBS (Dil), as a control, were injected intraperitoneally into Rag2^−/−^ mice. Animals receiving CNTRL splenocytes were used as internal control (Supplementary Figure 2). Mice were treated daily, beginning at day 7 after intravenous adoptive transfer of splenocytes. Administration was continued for 4 weeks and discontinued during the remaining 28 days follow up. To evaluate the efficacy of the drug we measured the PV score during time (Figures 3A,C, Supplementary Figure 2A). The DSC3 model responds very rapidly (day 14, *P* = 0.048) and seems to show a better recovery following m-PSL withdrawal (Figure 3A). On the contrary, the other pemphigus models (DSC3/DSG3 and DSG3) appear to be only partially responsive to m-PSL (Figures 3B,C). Indeed, in DSC3/DSG3 model m-PSL is statistically different from Dil only at day 14 (*P* = 0.025) (Figure 3B), while m-PSL treatment is not effective in DSG3 model (Figure 3C). PV score overtime was translated in AUC analysis. m-PSL treatment is statistically effective in reducing AUC compared to Dil only in DSC3 model (*P* = 0.01) (Figure 3G). PV score is partially supported by the evolution of mice weight during treatment (Figures 3D–F). In fact, this set of data shows that only the DSC3 model is capable of recovering weight following m-PSL administration, although no statistically significant differences between m-PSL and Dil group were observed. To evaluate the effect of m-PSL treatment on anti-DSC3 and anti-DSG3 antibody production, we performed ELISA assays during the administration and the follow-up period (Figure 3H, Supplementary Figure 3). This analysis shows that in DSC3 ELISA, m-PSL significantly suppresses the production of anti-DSC3 IgG starting at day 28 in DSC3 model (detailed in Supplementary Figure 3A), and at day 63 in DSC3/DSG3 mouse model (Supplementary Figure 3B), while no significant modulations were detected in DSG3 (Supplementary Figure 3C) and CNTRL (Supplementary Figure 2C) treated animals, as expected. In DSG3 ELISA, m-PSL significantly suppresses the production of anti-DSG3 IgG at day 63 in DSC3/DSG3 and DSG3 mouse models (detailed in Supplementary Figures 3E,F). No significant modulations were detected in DSC3 (Supplementary Figure 3D) and CNTRL (Supplementary Figure 2D) treated animals, as expected.

## Discussion

Pemphigus is a bullous disease classically associated with autoantibodies against DSG3 and DSG1 (21). Yet, in recent years, a number of non-desmoglein antibodies have been identified in serum from PV patients. These autoantibodies have been shown to play a role in keratinocyte adhesion (5, 6). At the same time, active mouse models able to mirror the different forms of pemphigus and in particular the variants involving the non-desmoglein antigens are still lacking. In the present study, we present two novel animal models of pemphigus developed by a protocol originally established for DSG3 PV (14). In particular, we have shown the contribution of DSC3 in the genesis of pemphigus. To our knowledge, this is the first pemphigus model generated by DSC3 antibodies.

In order to develop the DSC3 and the mixed DSC3/DSG3 models, Rag2^−/−^ mice were infused with splenocytes deriving from Dsg3^−/−^ mice immunized against recombinant DSG3 as described in literature (14) and/or with splenocytes deriving from WT mice that underwent breaking of immunological tolerance against DSC3. This approach was chosen since the DSC3-null mutation results in embryonic lethality, with most mutant embryos dying before they can implant into the uterus (22). Moreover, DSC3^fl/fl^/K14-Cre conditional null mice display very severe acantholytic phenotype (animals were not usually maintained more than 3 months of age), and approximately 10% of the newborn mutant mice, that developed severe epidermal blisters, die few hours after birth (19). Thus, immunological tolerance was broken by injecting WT mice with a recombinant protein obtained by baculovirus corresponding to the extracellular domain of DSC3 (Figure 1A), by modification of the protocol set-up by Hirose et al. (16). It should be taken into account that this kind of immunization strategy may only lead to a partial loss of tolerance, as compared to introducing a neo-antigen as in Dsg3^−/−^ mice immunized against rDSG3.

Desmocollins encode N-glycosylated transmembrane proteins that belong to the cadherin family of calcium-dependent cell-adhesion molecules. DSC3, in particular, is expressed mainly in the basal and first suprabasal cell layers of the skin and the presence of anti-DSC3 antibodies has already proved to be pathogenic (5, 6, 9, 20, 23).

Mice expressing antibodies against DSC3 started to develop disease signs a few days following splenocytes transfer. Unlike the DSG3 and DSC3/DSG3 models, the main features of DSC3 mice consisted of an intense erythema. Patchy hair loss and crusted lesions appeared to be milder than those observed in DSG3 and DSC3/DSG3 mice. The involvement of hair follicle is due to the specific expression of DSC3 and DSG3 molecules also in the bulge. In particular, DSC3 is the main DSC isoform synthesized in the basal and first suprabasal cell layers of the interfollicular epidermis and the outer root sheath of the hair follicle (19, 24). Moreover, DSC3 is required to maintain cell adhesion and hair follicle anchorage to the epidermis (19). DSG3 displays an expression pattern totally similar to DSC3. Indeed, DSG3 is not only critical for cell adhesion in the deep stratified squamous epithelium, but also for anchoring the telogen hair to the outer root sheath of the follicle, underscoring the importance of desmosomes in maintaining the normal structure and function of hair (25). Moreover, Kock and co-workers demonstrated that DSG3^−/−^ animals show defective cell-cell adhesion and hair loss (26). Lastly, Rag2^−/−^ mice actively producing anti-DSG3 antibodies displayed alopecia (14). All in all, the DSC3 phenotype may resemble that observed in so-called atypical pemphigus in humans where clinical presentation is always milder than in PV or PF (23, 27). Histologic analysis confirms the presence of acantholysis in DSC3 mice, though to a lower extent, as compared to the other models. The lower intensity of the symptoms developed by DSC3 mice might depend on the fact that DSC3 plays a major role in the desmosomes during embryo development (22), while in the adult its role is widely replaced by desmogleins. Moreover, it is likely that Ab titer could be important in determining the development of the skin manifestations. Indeed, Amagai and co-workers demonstrated that animals with low Ab production did not develop any pemphigus signs unless they were boosted with recombinant DSG3 (14). Here DSC3 animal model was not boosted with rDSC3, and, although the DSC3 Ab titer is lower than that of anti-DSG3 Ab, it is statistically modulated overtime and in the different models, indicating that phenotypic effects could be ascribed to this modulation. In addition, given that cadherins are adhesion molecules serving also as signaling mediators (28), the main difference between our active DSC3 mouse model and the DSC3^fl/fl^/K14-Cre conditional null mouse reported by Chen and co-workers could be explained by the different signaling activated by the presence of auto-Ab against DSC3, that is distinctive from the complete structural lacking of DSC3 expression (19). Future studies will evaluate the signaling activated by anti-DSC3 Auto-Abs. Lastly, DSC3 could be compensated by DSC2, which partially overlaps with DSC3 expression (19). In any event, the mild phenotype observed in our DSC3 mouse model is suggestive of the human clinical findings associated with atypical pemphigus (9). The mixed DSC3/DSG3 and DSG3 mice are clinically similar, consistent with the model described by Amagai and co-workers (14). However, we identified distinctive histological signs in the DSC3/DSG3 mouse model. In particular, we found occasional dermal eosinophils and epidermal focal spongiosis. Moreover, a mild inflammatory infiltrate was detected in the dermis, in the presence or absence of crusted lesions. These aspects are peculiar of the DSC3/DSG3 model, given that no significant infiltration of inflammatory cells was observed in the early stages of developing blisters in the DSG3 active pemphigus mouse model (14), indicating the uniqueness of our model and a cooperation of the different auto-Abs in the induction of the pemphigus phenotype (5, 6). On the other hand, this might also suggest that the role of DSC3 alone can only account for a milder form of pemphigus.

To further validate the DSC3 model, the three groups of animals underwent m-PSL treatment to verify and compare the responsiveness to this drug. DSG3 and DSC3/DSG3 pemphigus models only partially responded to therapy, while m-PSL was very efficacious in improving the DSC3 model, as shown by PV Score and DSC3 antibody titer. The differential response to m-PSL suggests that every model has its specificity and that the development of animals expressing auto-antibodies against different antigens might indeed be useful in order to preliminary verify which therapy might be more appropriate to target specific forms of pemphigus, characterized by specific subsets of autoantibodies.

The animal model presented here can open the way to the generation of active pemphigus models based on different antibodies.

## Data Availability

The datasets generated for this study are available on request to the corresponding author.

## Ethics Statement

Animal studies and animal procedures were approved by the Animal Welfare Committee of the University of Modena and Reggio Emilia and carried out in accordance with the Italian Institute of Health guidelines. The protocol was approved by the Italian Ministry of Health.

## Author Contributions

RL, TZ, and CP co-conceived this study and designed the experiments. RL, CA, LR, and GD performed the experiments and analyzed the data. RL, CA, AM, TZ, and CP wrote the manuscript and prepared the table/figures. All authors read and approved the final manuscript and agreed to submit it for publication.

### Conflict of Interest Statement

The authors declare that the research was conducted in the absence of any commercial or financial relationships that could be construed as a potential conflict of interest.
