# Supplementary material for: Development of a Desmocollin-3 Active Mouse Model Recapitulating Human Atypical Pemphigus
Source: Front Immunol. 2019 Jun 19;10:1387. doi: 10.3389/fimmu.2019.01387 (PMC6593104; doi:10.3389/fimmu.2019.01387)
Supplement: Supplementary file 1 [file Data_Sheet_1.PDF]

Supplementary Material

1 Supplementary Figures

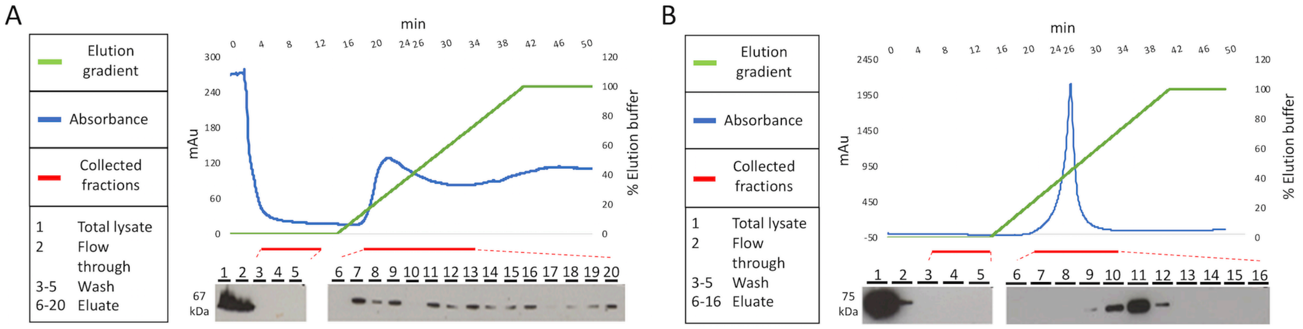

Supplementary Figure 1. Affinity purification of murine recombinant proteins. (**A**) DSC3 and (**B**) DSG3 schematic representations of recombinant protein purification (chromatograms), with western blot controls of each fractions (anti-His-tag Ab).

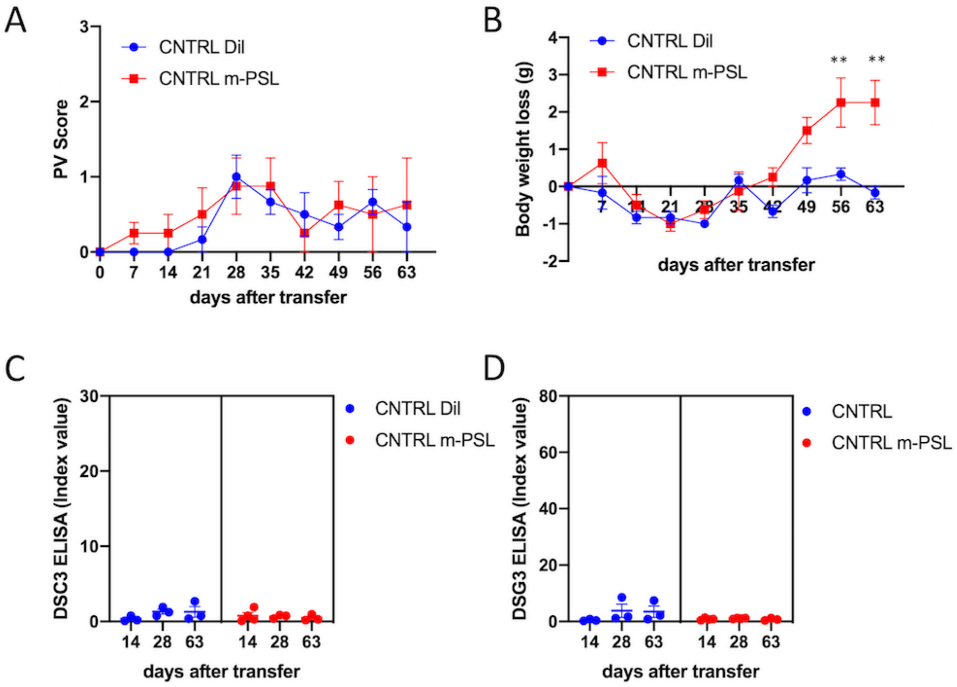

Supplementary Figure 2. Effect of methyl-prednisolone (m-PSL) on CNTRL mice. m-PSL was administered i.p. daily from day 7 after the adoptive transfer to day 35. CNTRL animals were randomly assigned to the m-PSL or PBS (Dil) treatment group (n=5 animals per group). PV score (A) and body weight variations (B) were reported weekly, till day 63. (**A**) PV score: no statistically significant differences among m-PSL and Dil group. (**B**) Body weight loss: m-PSL induced a statistically significant increase in body weight if compared to Dil group, at day 56 (P=0.0056) and day 63 (P=0.0003). (**C**) DSC3 ELISA assay and (**D**) DSG3 ELISA assay: by multiple t-test, no statistically significant differences among m-PSL and Dil group.

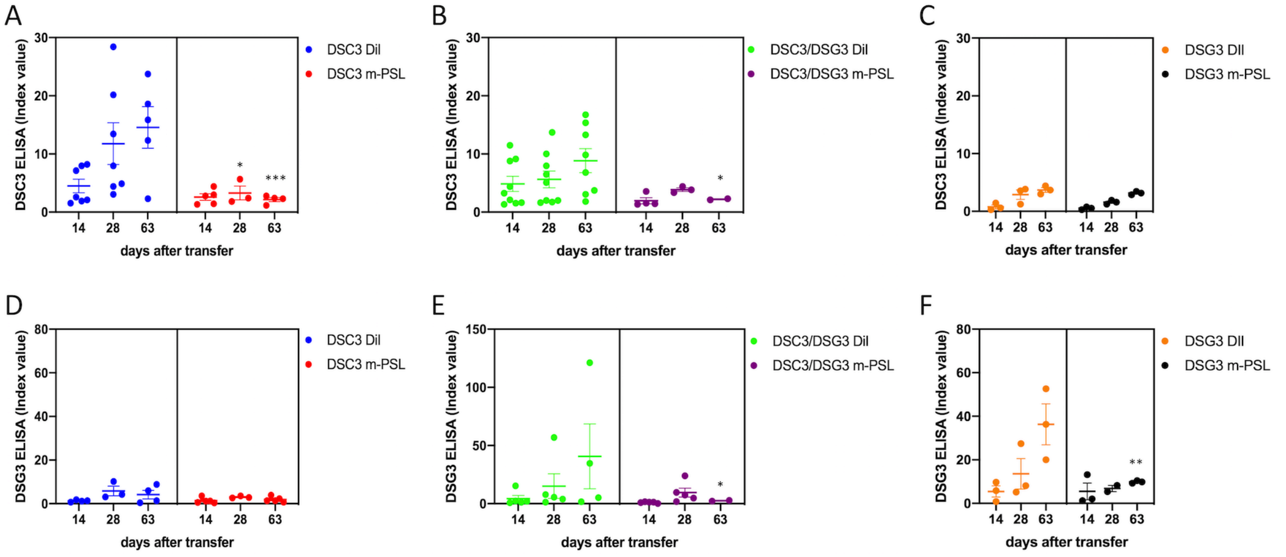

Supplementary Figure 3. Effect of m-PSL treatment on the autoantibody production in the three pemphigus mouse models. (**A-B-C**) DSC3 ELISA. Two-way ANOVA between conditions, P<0.0001. (**A**) In DSC3 model, m-PSL significantly suppressed the production of anti-DSC3 IgG starting from day 28, P=0.0329, till day 63, P=0.0002 (Multiple t-test). (**B**) In DSC3/DSG3 mouse model, m-PSL significantly suppressed the production of anti-DSC3 IgG at day 63, P=0.022 (Multiple t-test). (**C**) In DSG3 mouse model, no statistically significant differences among m-PSL and Dil group at any timepoint. (**D-E-F**) DSG3 ELISA assay. Two-way ANOVA between conditions P=0.0076. (**D**) In DSC3 model, no statistically significant differences among m-PSL and Dil group at any timepoint. (**E**) In DSC3/DSG3 model, m-PSL significantly suppressed the production of anti-DSG3 IgG at day 63, P=0.042 (Multiple t-test). (**F**) In DSG3 mouse model, m-PSL significantly suppressed the production of anti-DSG3 IgG at day 63, P=0.0027 (Multiple t-test).
